# How useful is a history of rubella vaccination for determination of disease susceptibility? A cross-sectional study at a public funded health clinic in Malaysia

**DOI:** 10.1186/1471-2296-14-19

**Published:** 2013-01-31

**Authors:** Ai Theng Cheong, Seng Fah Tong, Ee Ming Khoo

**Affiliations:** 1 Department of Family Medicine, Faculty of Medicine and Health Sciences, Universiti Putra Malaysia, Serdang, Selangor, 43400, Malaysia; 2 Department of Family Medicine, Faculty of Medicine, Universiti Kebangsaan Malaysia, Jalan Yaacob Latif, Cheras, Kuala Lumpur, 56000, Malaysia; 3 Department of Primary Care Medicine, Faculty of Medicine, University of Malaya, Kuala Lumpur, 50603, Malaysia

**Keywords:** Rubella susceptibility, History, Vaccination, Sensitivity, Specificity

## Abstract

**Background:**

Identification of pregnant women susceptible to rubella is important as vaccination can be given postpartum to prevent future risks of congenital rubella syndrome. However, in Malaysia, rubella antibody screening is not offered routinely to pregnant women in public funded health clinics due to cost constraint. Instead, a history of rubella vaccination is asked to be provided to establish the women’s risk for rubella infection. The usefulness of this history, however, is not established. Thus, this paper aimed to determine the usefulness of a history of rubella vaccination in determining rubella susceptibility in pregnant women.

**Methods:**

A cross-sectional study was conducted on 500 pregnant women attending a public funded health clinic. Face-to-face interviews were conducted, and demographic data and history of rubella vaccination were obtained. Anti-rubella IgG test was performed.

**Results:**

A majority of the women (66.6%) had a positive vaccination history. Of these, 92.2% women were immune. A third (33.4%) of the women had a negative or unknown vaccination history, but 81.4% of them were immune to rubella. The sensitivity and specificity of a history of rubella vaccination in identifying disease susceptibility was 54.4% (95% CI: 40.7, 67.4%) and 69.3% (95% CI: 64.7, 73.5%) respectively; the positive predictive value was 18.6% (95% CI: 13.1, 25.5%) and the negative predictive value was 92.2% (95% CI: 88.6, 94.7%).

**Conclusions:**

A vaccination history of rubella had a poor diagnostic value in predicting rubella susceptibility. However, obtaining a vaccination history is inexpensive compared with performing a serological test. A cost-utility analysis would be useful in determining which test (history versus serological test) is more cost-effective in a country with resource constraint.

## Background

Rubella infection is a mild disease when it affects children. However, when it affects pregnant women in the early trimester, it can cause serious complications such as miscarriage to the mother and congenital rubella syndrome (CRS) to the fetus [1,2]. CRS is an important cause of severe birth defects with ophthalmic, auditory, cardiac and neurological abnormalities.

Although some women may acquire natural immunity against rubella by virtue of being infected in childhood, which is often asymptomatic [3], it is still important to vaccinate susceptible women as CRS carries serious consequences.

Cutts et al. (1999) estimated a total of 110,000 CRS cases in the developing countries [4]. The incidence rate of CRS in developing countries ranged from 0.4 to 4.3 per 1000 live births [5]. In Malaysia, a retrospective review of rubella serology from 1993–1998 at University Hospital, Kuala Lumpur had reported an incidence rate of 0.5 per 1000 live births for CRS [5,6]. In recent years, sporadic cases of CRS have been reported in literature [7,8], although the exact number is unknown.

One of the ways to reduce CRS is to vaccinate all women before they reach reproductive age. The World Health Organization (WHO) has reported that 131 out of 193 WHO member countries (68%) have included rubella vaccination in their national immunization schedule [9]. Malaysia started the rubella vaccination program in 1988 targeting school girls aged 12 to 15 years, and women in the reproductive age group (15 – 44 years) [10]. Since 2002, the program has replaced rubella vaccine with the 2-dose measles-mumps-rubella (MMR) vaccination for all children aged 1 and 7 years [11]. In 2011, the uptake of the MMR vaccination for children aged 1 to 2 years was about 95% [12]. Despite the vaccination program, outbreak of rubella has been reported among 16-year old students in a military vocational training school in 2006 [13] and the prevalence of rubella susceptibility in pregnant women has been reported to range between 8% and 11% in Malaysia [14,15]. This prevalence is relatively high compared with some countries in the Asia-pacific region such as Australia (2.7%) [16] and Japan (6.7%) [17], but is lower than Singapore (15.8%), Thailand (18.0%), Taiwan (16.7%) and Sri Lanka (24%) [18-21]. Therefore, efforts are still needed to reduce rubella susceptibility among pregnant women.

To achieve a reduction in rubella susceptibility in women, apart from implementing rubella vaccination as part of the childhood immunization schedule, supplementary vaccination can be employed as an additional strategy [22]. In some countries, screening for rubella antibodies is carried out during antenatal period to identify susceptible women who are then vaccinated post partum [22-24]. In Malaysia, rubella antibody is not routinely screened for in pregnant women in public funded health clinics due to cost constraint. Instead, these women are routinely asked for a history of rubella vaccination to identify their risk for rubella infection as some studies have shown that rubella vaccination status is significantly associated with susceptibility to rubella infection [20,21]. Women who have no history of rubella vaccination are then offered the vaccination post partum. The aim of this study was thus to determine the usefulness of a history of rubella vaccination as a screening tool for rubella susceptibility in countries such as Malaysia that do not offer routine antenatal rubella antibody screening.

## Methods

### Study design and participants

A cross-sectional study was carried out at a public funded health clinic from June to October 2005 in the state of Selangor, Malaysia. All pregnant women aged 15 to 45 years attending the clinic for antenatal booking were invited to participate in the study. Women with symptoms of possible acute rubella infection as signified by fever and rash were excluded from the study. Pregnant women who consented to the study were recruited. For women aged less than 18 years, written consents were taken from both the women and their guardians. The study was approved by the Medical Ethics Committee of University Malaya Medical Centre and the Ministry of Health, Malaysia.

### Setting

Malaysia is a multiracial country with a population of 28.3 million according to the population census of 2010. The ethnic distribution consists of 67.4% Malay, 24.6% Chinese, 7.3% Indian and 0.7% others [25]. Selangor is the most densely populated among the fourteen states of the country. It has a population of 5.46 million and is highly urbanized. The health clinic in this study served a population of 67,578 [25]. It has a maternal and child health clinic that provides free service to Malaysian citizens. The ethnic distribution of this study-population of female aged 15–45 years was similar to the Selangor and Malaysian census [25,26].

### Data collection

Face-to-face interviews were conducted using structured data collection forms to obtain socio-demographic data, history of rubella vaccination and past history of rubella infection. (Additional file 1). For history of rubella vaccination, the following question was asked: “Have you ever been vaccinated for rubella?” There were three options for response: “yes”, “no” or “don’t know”. Those who answered “no” or “don’t know” were considered susceptible to rubella infection. The history of rubella vaccination was self reported as there was no documentation of vaccination available for verification. The women were also asked for past history of rubella infection. Those who answered yes were asked if they had the diagnosis verified either by a doctor or by a blood test.

All recruited women then had their serum IgG rubella antibody level measured, and this level was used as the gold standard for rubella susceptibility. The IgG rubella antibody was measured using the AxSYM Rubella IgG assay based on the Microparticle Enzyme Immunoassay (MEIA) technology. A rubella IgG antibody level of 10 IU/mL or greater indicates immunity to rubella infection either through previous rubella infection or induced by previous vaccine. A rubella IgG antibody level of less than 10 IU/mL is considered susceptible to rubella infection.

### Sample size

Using epi-info version 6.0, the sample size was calculated based on the expected prevalence of rubella susceptibility of 8% and the worst acceptable prevalence of 11% with a confidence level of 95%. The estimated sample size was 314.

### Statistical analysis

Data were analyzed using SPSS version 15. Sensitivity and specificity, the positive and negative predictive values of a history of rubella vaccination in identifying rubella susceptibility were determined using anti-rubella IgG level as the gold standard. In this study, the “test” was a “history of rubella vaccination”. The aim of the “test” was to correctly identify women who were susceptible to rubella. The gold standard for rubella susceptibility was those with anti-rubella IgG<10 IU/mL. Sensitivity of the test is defined as the percentage of all susceptible women who indeed reported to be not vaccinated or to be unknown whether vaccinated. Specificity is defined as the percentage of all protected women that indeed reported to be vaccinated. Positive predictive value of the test is the chance that a woman who reported to be not vaccinated was susceptible to rubella.

## Results

Five hundred and two (502) patients who satisfied the inclusion criteria were approached. Two patients refused to participate. The response rate was 99.6%. A majority of women were Malay (55.2%), followed by Chinese (23.0%), Indian (8.6%) and others (13.2%). The age ranged from 16 to 42 years, the mean age being 27.2 (SD 4.8) years; the median age was 27.0 years (IQR 6.0). Half the women were primigravida (49.2%). None of them reported a past history of rubella infection that was confirmed by clinical diagnosis of a physician or by blood test. The prevalence of rubella susceptibility in this study was 11.4% [95% CI: 8.6, 14.2%].

Most of the women (66.6%) had a positive vaccination history. Of these, 92.2% were immune. (Table 1) A third (33.4%) of the women had a negative vaccination history (no history of previous vaccination or unknown vaccination status); nevertheless, 81.4% of them were immune to rubella. (Table 2) A history of rubella vaccination was significantly associated with rubella susceptibility (χ^2^ = 12.737, P ≤0.001). Logistic regression results from our previous analysis have shown that a negative vaccination history was a significant predictor for rubella susceptibility (odd ratio=2.7, 95% CI: 1.5, 4.7) after controlling for age, ethnicity, parity, education level and occupation [15].

**Table 1:** Self reported rubella vaccination history and rubella susceptibility

**Self reported rubella vaccination history**	**Rubella susceptibility(anti**-**rubella IgG test)**		**Total N=500 N(%)**
	**Susceptible(IgG<10 IU**/**mL)N=57 N(%)**	**Non-susceptible(IgG≥10 IU/mL)N=443 N(%)**	
No history of vaccination	22 (33.3)	44 (66.7)	66 (100.0)
Unknown vaccination status	9 (8.9)	92 (91.1)	101 (100.0)
Vaccinated	26 (7.8)	307 (92.2)	333 (100.0)

**Table 2:** **Sensitivity**, **specificity**, **positive and negative predictive value of rubella vaccination history against rubella susceptibility**

**Rubella vaccination history**	**Rubella susceptibility**		**Total(%)**
	**(anti**-**rubella IgG test)**		
	**Susceptible(IgG<10 IU/mL)N(%)**	**Immune(IgG≥10 IU/mL**) **N(%)**	
No history of vaccination or unknown vaccination status ( rubella susceptible)	31 (18.6%)	136 (81.4%)	167 (100%)
History of vaccination(rubella immuned)	26 (7.8%)	307 (92.2%)	333 (100%)
Total	57	443	500

Sensitivity of rubella vaccination history for rubella susceptibility= 31/ (31+26 ) x 100 = 54.4% (95% CI: 40.7, 67.4%).

Specificity of rubella vaccination history for rubella susceptibility= 307/ (307+136) x 100 = 69.3% (95% CI: 64.7, 73.5%).

Positive predictive value of rubella vaccination history = 31/ (31+136) x 100 = 18.6% (95% CI: 13.1, 25.5%).

Negative predictive value of rubella vaccination history = 307/ (307+26) x 100 = 92.2% (95% CI: 88.6, 94.7%).

The sensitivity of a history of rubella vaccination in identifying rubella susceptibility using serological test as gold standard was 54.4% (95% CI: 40.7, 67.4%) and the specificity was 69.3% (95% CI: 64.7, 73.5%). The positive predictive value was 18.6% (95% CI: 13.1, 25.5%) and the negative predictive value was 92.2% (95% CI: 88.6, 94.7%) (Table 2).

## Discussion

The aim of the test (vaccination history) was to identify women susceptible to rubella in order to offer them vaccination to prevent rubella in future pregnancies. For this purpose, sensitivity is the most important element of the test. The sensitivity of the test was 54%. Hence, on the basis of just a history of rubella vaccination, almost half the susceptible women would be overlooked and remain unprotected against rubella. On the other hand, compared with no serological testing at all, a negative vaccination history would at least identify half of the susceptible women. The specificity of the test was 69%; however, it is not a clinical problem that 31% of the women were protected while they reported to be not vaccinated. This could be due to natural immunity they acquired during childhood subclinical infection that often goes unnoticed. The positive predictive value of the test is only 19%, which means that there is a one in five chance that a woman who reported to be not vaccinated was susceptible to rubella, the majority being protected. The negative predictive value was 92%, which means that the majority of women who reported positive vaccination history were protected. The relatively low sensitivity and positive predictive value of the rubella vaccination history might not be useful in screening for rubella-susceptible women. Serological testing, the gold standard test, is superior in correctly identifying women who are susceptible to rubella. Nonetheless, serological test is expensive compared with an enquiry of vaccination history. A cost-utility analysis is needed to determine which test is preferable from a public health point of view: a history of vaccination “test” or a serological test, especially for a resource constrained country.

7.8% of the women who reported having had vaccination were found to have low antibody titer level and were considered to be not protected based on the cut-off level of anti-rubella IgG of 10 IU/mL. This proportion is substantial given the serious complications resulting from congenital rubella syndrome. Therefore, screening for rubella susceptibility using a history of rubella vaccination would have overlooked this group of women and denied them the benefit of rubella booster dose to prevent congenital rubella syndrome. Some studies have reported the possibility of a gradual decline in the rubella antibody titer from a protective level to a non-protective level in some individuals who had the vaccination [27-29]. Therefore, it is possible for rubella to occur during pregnancy even in women who have had the vaccination.

About two-third of the women who reported negative rubella vaccination history had protective antibody titers. Some of these women may have acquired the immunity through subclinical infections they were not aware of when they were young [3]. Some women may have experienced fever and rash and did not relate it to rubella infection as these symptoms mimic other common viral infections. Locally, rubella antibody test is not performed in women for confirmation of the disease as the disease is mild and self-limiting, and does not require notification.

In this study, we used anti-rubella IgG level of 10 IU/mL as the cut-off point for protection against rubella infection. This was the recommended level based on earlier epidemiological studies when rubella infection was prevalent [30]. Although a recent study on the sero-prevalence of rubella antibody over time has suggested that the cut-off level of anti-rubella IgG for protection against infection should be adjusted according to an immunized antenatal population rather than following the older recommendation [30], we lacked local serological data regarding rubella antibody level for women of childbearing age to ascertain the level of protection for rubella infection. Until such data becomes available, the cut-off level of 10 IU/mL would be used.

The strength of this study was the recruitment of participants from a public funded health clinic, which provided most of the antenatal care for pregnant women in the country. The study is limited by its cross-sectional design and the socio-economic status of the participants, who were mainly from the middle and lower socio-economic groups that may differ from patients seen in other settings. Therefore, caution is necessary in generalizing the findings. Nevertheless, this study that has provided us the preliminary evidence of an inexpensive test, namely, a history of rubella vaccination, is not a useful screening tool for rubella susceptibility. Further study is needed to confirm this as it may have implications on the healthcare policy regarding rubella antibody testing in pregnant women.

## Conclusions

A test based on the history of rubella vaccination has low sensitivity, specificity and predictive values in determining rubella susceptibility. Serological testing needs to be considered in all women of childbearing age to effectively prevent the occurrence of congenital rubella syndrome. However, as serology test is more expensive than taking a vaccination history, a cost-utility analysis is recommended in case of resource constraint.

## Competing interests

The authors declare no competing interests.

## Authors’ contributions

All authors contributed to the design, analysis and write up of this study. ATC produced the first draft of the manuscript. All authors contributed to the revision of the draft. The final manuscript has been read and approved by all authors.

## Pre-publication history

The pre-publication history for this paper can be accessed here:

http://www.biomedcentral.com/1471-2296/14/19/prepub

## Supplementary Material

Addistional file 1 Structured data collection form. Click here for file
