# Supplementary material for: How useful is a history of rubella vaccination for determination of disease susceptibility? A cross-sectional study at a public funded health clinic in Malaysia
Source: BMC Fam Pract. 2013 Jan 31;14:19. doi: 10.1186/1471-2296-14-19 (PMC3564688; doi:10.1186/1471-2296-14-19)
Supplement: Addistional file 1 — Structured data collection form. [file 1471-2296-14-19-S1.pdf]

Additional File 1. Structured data collection form

Date:

RN:
Contact No:
I/C No:

Demographic data

1. Age:

2. Parity:

3. Period of amenorrhea:

4. Race:

Malay	
Chinese	
Indian	
Indonesian	
Others	

5. Citizens: Malaysian/Foreigners from_______________(country)

6. Education level:

No formal education	
Primary	
Secondary	
Higher education	

7. Occupation:

8. Household incomes: RM……………………………

History

1. Rubella vaccination before:

yes	
no	
don't know	

2. Past history of German Measles/ Rubella infection?

yes		yes only for those had been told by doctor clinically or confirmed by blood test
no		
don't know		

3. Past history of contact with people/family members with German Measles/Rubella?

yes		yes if the contacts had been told by doctor about the diagnosis or confirmed by blood test
no		
don't know		

Laboratory test

Rubella IgG titre:
